# Japanese classification of pancreatic carcinoma by the Japan Pancreas Society: Eighth edition

**DOI:** 10.1002/jhbp.12056

**Published:** 2024-07-29

**Authors:** Masaharu Ishida, Tsutomu Fujii, Masashi Kishiwada, Kazuto Shibuya, Sohei Satoi, Makoto Ueno, Kohei Nakata, Shigetsugu Takano, Katsunori Uchida, Nobuyuki Ohike, Yohei Masugi, Toru Furukawa, Kenichi Hirabayashi, Noriyoshi Fukushima, Shuang‐Qin Yi, Hiroyuki Isayama, Takao Itoi, Takao Ohtsuka, Takuji Okusaka, Dai Inoue, Hirohisa Kitagawa, Kyoichi Takaori, Masaji Tani, Yuichi Nagakawa, Hideyuki Yoshitomi, Michiaki Unno, Yoshifumi Takeyama

**Affiliations:** ^1^ Department of Surgery Tohoku University Graduate School of Medicine Sendai Japan; ^2^ Department of Surgery and Science, Faculty of Medicine Academic Assembly, University of Toyama Toyama Japan; ^3^ Department of Hepatobiliary Pancreatic and Transplant Surgery Mie University Hospital Tsu Japan; ^4^ Department of Surgery Kansai Medical University Hirakata Japan; ^5^ Department of Gastroenterology Kanagawa Cancer Center Yokohama Japan; ^6^ Department of Surgery and Oncology, Graduate School of Medical Sciences Kyushu University Fukuoka Japan; ^7^ Department of General Surgery Chiba University Graduate School of Medicine Chiba Japan; ^8^ Department of Pathology Mie University Hospital Tsu Japan; ^9^ Department of Pathology St. Marianna University School of Medicine Kawasaki Japan; ^10^ Division of Diagnostic Pathology Keio University School of Medicine Tokyo Japan; ^11^ Department of Investigative Pathology Tohoku University Graduate School of Medicine Sendai Japan; ^12^ Department of Diagnostic Pathology, Faculty of Medicine Academic Assembly, University of Toyama Toyama Japan; ^13^ Department of Pathology Jichi Medical University Shimotsuke Japan; ^14^ Department of Frontier Health Sciences, Graduate School of Human Health Sciences Tokyo Metropolitan University Tokyo Japan; ^15^ Department of Gastroenterology, Graduate School of Medicine Juntendo University Tokyo Japan; ^16^ Department of Gastroenterology and Hepatology Tokyo Medical University Tokyo Japan; ^17^ Department of Digestive Surgery, Graduate School of Medical and Dental Sciences Kagoshima University Kagoshima Japan; ^18^ Department of Hepatobiliary and Pancreatic Oncology National Cancer Center Hospital Tokyo Japan; ^19^ Department of Radiology Kanazawa University Graduate School of Medical Sciences Kanazawa Japan; ^20^ Department of Surgery Kurashiki Central Hospital Kurashiki Japan; ^21^ Department of Surgery Nagahama City Hospital Nagahama Japan; ^22^ Department of Surgery Shiga University of Medical Science Otsu Japan; ^23^ Department of Gastrointestinal and Pediatric Surgery Tokyo Medical University Tokyo Japan; ^24^ Department of Surgery Dokkyo Medical University Saitama Medical Center Koshigaya Japan; ^25^ Department of Surgery Kinki University School of Medicine Osakasayama Japan

**Keywords:** classification, Japanese, neoplasm staging, pancreatic cancer, pancreatic neoplasms

## Abstract

In 2023, the Japan Pancreas Society (JPS) published the new eighth edition of the Japanese classification of pancreatic carcinoma. We present here an excerpted version in English, based on the latest edition. The major changes in this revision are as follows: In the eighth edition of the Union for International Cancer Control (UICC), the T category was changed to be based on tumor size; however, the eighth edition of the Japanese classification retains the previous T category based on local invasion factors. Lymph nodes have been renamed, and regional lymph nodes have been defined by location. Peritoneal cytology, which was not previously included in distant metastasis (M), has now been included in the M category. Moreover, significant additions have been made regarding the pathological diagnosis of endoscopic ultrasound‐guided fine‐needle aspiration biopsy (EUS‐FNAB) and criteria for histological assessment of the effects after chemotherapy and radiation therapy. Although this classification is aimed at carcinoma originating in the pancreas, not in the bile duct or duodenum, if the differentiation of the primary organ is difficult, this classification should be applied. It is also desirable to describe tumors other than carcinoma and metastatic tumors to the pancreas in accordance with this classification.

## Graphical abstract

Ishida and colleagues report that the eighth edition of the Japanese classification of pancreatic carcinoma retains the T category, introduces new lymph node names, incorporates peritoneal cytology into the M category, and addresses endoscopic ultrasound‐guided fine‐needle aspiration biopsy and post‐therapy effects. It is also valuable for characterizing tumors other than carcinoma and metastatic tumors in the pancreas.

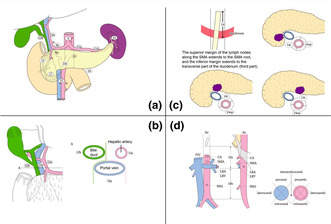

## 1 INTRODUCTION

The first edition of the Japanese classification of pancreatic carcinoma was published in 1980 by the Japan Pancreas Society (JPS)^1^ and has been revised seven times, with the eighth edition being the most recent.^2^ The seventh edition (2016) included revisions that maintained its uniqueness and established consistency with the seventh edition (2009) of the Union for International Cancer Control (UICC).^3^

While the eighth edition of the UICC classification has changed the T category to be based on tumor size, the eighth edition of the JPS classification maintains the previous T category based on local invasion. Major changes in the TNM classification include revisions of the station numbers and names of lymph nodes, defining regional lymph nodes by tumor location, and including positive peritoneal cytology, which was not previously included in distant metastasis but is now considered distant metastasis.^4^

The purpose of cancer classification is to establish clinical and pathological management strategies to enable clinicians and pathologists to compare and discuss the collected data and clinical outcomes based on common criteria, with the ultimate goal of improving the treatment outcomes of pancreatic cancer.

## 2 GENERAL PRINCIPLES OF RECORDING FINDINGS

The main disease in this classification is primary pancreatic carcinoma. Therefore, cancers arising in the pancreatic bile duct, duodenum, or duodenal papilla are not included. However, if differentiation is difficult, these cancers may be managed according to this classification.

Categories of findings are abbreviated by using uppercase letters: “T” for the extent of invasion by the primary tumor, “N” for lymph node metastasis, and “M” for distant metastasis. The grade of each finding is indicated by Arabic numerals following the uppercase letter for each category, and “X” is used when the finding is unknown. The stage is recorded using a combination of T, N, and M findings. The findings at the time of diagnosis, namely, the clinical, surgical, pathological, and final findings, are indicated by the lower‐case letters “c,” “s,” “p,” and “f,” respectively. The symbols “m,” “y,” “r,” and “a” may also be used to identify special cases in the TNM classification systems, representing multiple, yield to treatment, recurrent, and autopsy, respectively.

## 3 DESCRIPTION OF FINDINGS

### 3.1 Primary tumor

#### 3.1.1 Tumor location

**FIGURE 1:**
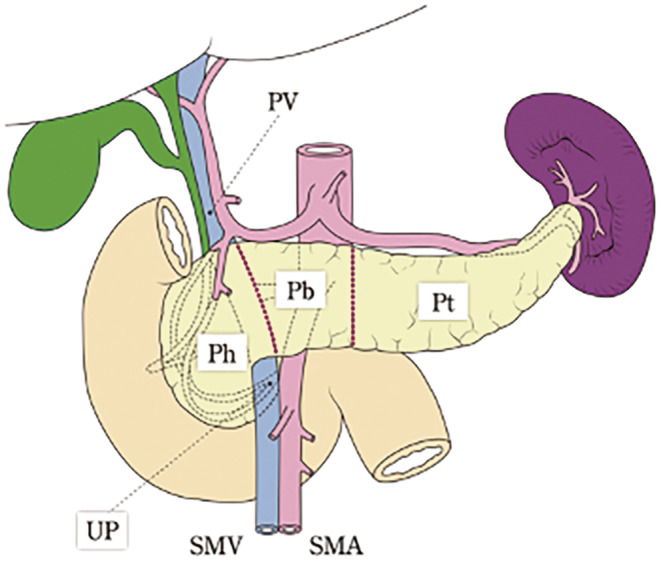
Anatomical portions of the pancreas. Reprinted with permission from the Japan Pancreas Society and Kanehara & Co., Ltd.: Japanese Classification of Pancreatic Carcinoma, eighth edition, 2023. The border between the pancreatic head and body is defined as the left side of the SMV and PV. The neck of the pancreas (the part anterior to the SMV and PV) and the uncinate process are included in the pancreatic head. The border between the pancreatic body and tail is defined as the left border of the abdominal aorta. Pb, pancreatic body; Ph, pancreatic head; Pt, pancreatic tail; PV, portal vein, SMA, superior mesenteric artery; SMV, superior mesenteric vein; UP, uncinate process.

The pancreas is anatomically divided into three main parts: head, body, and tail. The uncinate process is located in the pancreatic head. When a lesion spans more than two adjacent parts, the primary region should be described first, followed by the additional portion involved in infiltration (Figure 1).

#### 3.1.2 Size and number of the tumors

The largest diameter (mm) of the primary tumor is recorded as the tumor size (TS). Based on this, the tumor size is further classified as follows:

- TS1: Tumor size of 20 mm or less in the largest diameter
- TS2: Tumor size >20 mm but not >40 mm in the largest diameter
- TS3: Tumor size >40 mm but not >60 mm in the largest diameter
- TS4: Tumor size greater than 60 mm

For multiple tumors, the number, location, and largest diameter of the tumors are recorded.

#### 3.1.3 Macroscopic type

- Masked type: No macroscopic evidence of tumor
- Nodular type: Tumor with a clearly defined border
- Infiltrative type: Tumor with a poorly defined border and diffuse infiltration into the surrounding tissue
- Cystic type: Cystic tumors, such as cystadenocarcinoma
- Ductectatic type: Tumor with pancreatic duct dilation as a dominant feature
- Mixed type: A mixture of two or more macroscopic types
- Unclassified type: Tumor that cannot be classified into any of the above types

#### 3.1.4 Grade of local invasion (T category)

The definition of T categories for primary pancreatic cancer in the JPS eighth edition is different from that in the UICC eighth edition. In particular, T1 to T3 is unique in that the JPS eighth edition evaluates the category by combining “tumor size” and “extrapancreatic extension,” which is defined as a “local invasion factor,” while the UICC eighth edition defines the category based on “tumor size” alone and does not consider “extrapancreatic extension.” Additionally, T4 is based on celiac artery (CA) or superior mesenteric artery (SMA) invasion, whereas T1 tumors are subcategorized into T1a, T1b, and T1c based on their sizes.

- TX: Local invasion cannot be assessed
- T0: No evidence of primary tumor
- Tis: Carcinoma in situ
- T1: Tumor limited to the pancreas, with a size of 20 mm or less in the largest diameter
  ⚬ T1a: Tumor size of 5 mm or less in the largest diameter
  ⚬ T1b: Tumor size greater than 5 mm and less than 10 mm in the largest diameter
  ⚬ T1c: Tumor size greater than 10 mm but no more than 20 mm in the largest diameter
- T2: Tumor limited to the pancreas, with a size of more than 20 mm in the largest diameter
- T3: Tumor extends beyond the pancreas but does not involve the CA or SMA
- T4: Tumor involves the CA or SMA

#### 3.1.5 Local invasion factors

- Bile duct invasion: CH0, absent; CH1, present; CHX, cannot be assessed.
- Duodenal invasion: DU0, absent; DU1, present; DUX, cannot be assessed.
- Serosal invasion: S0, absent; S1, present; SX, cannot be assessed.
- Retropancreatic tissue invasion: RP0, absent; RP1, present; RPX, cannot be assessed.
- Portal venous system invasion: PV0, absent; PV1, present; PVX, cannot be assessed.

The portal venous system consists of the portal vein (PVp), superior mesenteric vein (PVsm), and pancreatic vein (PVsp).

- Arterial system invasion: A0, absent; A1, present; AX, cannot be assessed.

The arterial system comprises the superior mesenteric artery (Asm), celiac artery (Ace), common hepatic artery (Ach), and splenic artery (Asp).

- Extrapancreatic nerve plexus invasion: PL0, absent; PL1, present*; PLX, cannot be assessed.

The extrapancreatic nerve plexus is explained in the next section.

- Invasion of other organs: OO0, absent; OO1, present; OOX, cannot be assessed.

The other organs include the adrenal gland, stomach, large intestine, spleen, renal vein, kidney, inferior vena cava, and aorta. The names of the invading organs should be clearly indicated.

#### 3.1.6 Anatomical reconsideration of the extrapancreatic nerve plexus

**FIGURE 2:**
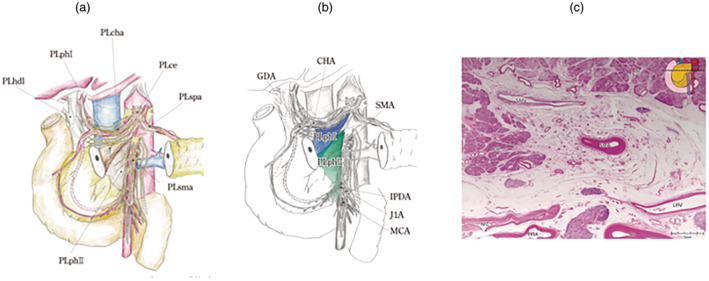
Extrapancreatic nerve plexus. Reproduced with permission from the Japan Pancreas Society and Kanehara & Co., Ltd.: Japanese Classification of Pancreatic Carcinoma, eighth edition, 2023. (a) The extra‐pancreatic nerve plexus is differentiated into seven categories (PLph I, pancreatic head nerve plexus I; PLph II, pancreatic head nerve plexus II; PLsma, superior mesenteric artery nerve plexus; PLcha, common hepatic artery nerve plexus; PLhdl, hepatoduodenal ligament nerve plexus; PLspa; splenic artery nerve plexus; PLce, celiac nerve plexus). (b) PLphI and PLphII are blue and green, respectively. (c) PLphII and PLsma are histologically examined in the anatomical samples. CHA, common hepatic artery; GDA, gastroduodenal artery; IPDA, inferior pancreatoduodenal artery; J1A, first jejunal artery; MCA, middle colic artery; SMA, superior mesenteric artery.

The definition and classification of the extrapancreatic nerve plexus are based on the work of Yoshioka et al., categorizing it into seven plexuses^5^, ^6^ (Figure 2a,b).

Figure 2c shows an anatomical specimen that was histologically examined for PLsma and PLph II. PLsma is characterized by concentric fibrous tissue centered around the SMA, with a thickness of approximately 3 mm. PLph II transitions naturally from PLsma, and there is no clear boundary, making it challenging to differentiate the two in pathological examinations. To achieve an accurate diagnosis of the infiltration site, it is desirable for the surgeon to precisely record the resected area and extent and immediately perform inking on the dissected and cut surfaces.

According to the National Comprehensive Cancer Network (NCCN) guidelines,^7^ the resection margin in the direction of the SMA is collectively referred to as the SMA (retroperitoneal/uncinate) margin, and the distance to the margin should be reported in millimeters because of its importance as a critical resection margin. In Japan, there is a history of evaluating soft tissues, such as PLph II and PLsma, where cancer infiltration is prone to occur. This system is not only useful for assessing the degree of infiltration and resection sites in both intraoperative findings and preoperative imaging diagnoses but also provides insights into the characteristics of pancreatic cancer progression, making it a forward‐thinking approach.

### 3.2 Lymph node metastases (N category)

#### 3.2.1 Station numbers, locations, and boundaries of lymph nodes related to the pancreas

Table 1, Figure 3.

**TABLE 1:** Station numbers and names of lymph nodes.

Number	Location
1	Right cardial lymph nodes
2	Left cardial lymph nodes
3	Lymph nodes along the lesser curvature of the stomach
4	Lymph nodes along the greater curvature of the stomach
5	Suprapyloric lymph nodes
6	Infrapyloric lymph nodes
7	Lymph nodes along the left gastric artery
8a	Lymph nodes in the anterosuperior group along the common hepatic artery
8p	Lymph nodes in the posterior group along the common hepatic artery
9	Lymph nodes around the celiac artery
10	Lymph nodes at the splenic hilum
11p	Lymph nodes along the proximal splenic artery
11d	Lymph nodes along the distal splenic artery
12a	Lymph nodes along the hepatic artery
12p	Lymph nodes along the portal vein
12b	Lymph nodes along the bile duct
13	Lymph nodes on the posterior aspect of the head of the pancreas
14t^a^	Lymph nodes along the superior mesenteric artery (tumor side)
14op^a^	Lymph nodes along the superior mesenteric artery (opposite side of the tumor)
15	Lymph nodes along the middle colic artery
16a	Lymph nodes around abdominal aorta a
16b	Lymph nodes around abdominal aorta b
17	Lymph nodes on the anterior surface of the head of the pancreas
18	Lymph nodes along the inferior margin of the pancreas

*Note*: Reproduced with permission from the Japan Pancreas Society and Kanehara & Co., Ltd.: Japanese Classification of Pancreatic Carcinoma, eighth edition, 2023.

^a^ The extent of lymph node 14 is defined as up to the lower margin of the transverse part of the duodenum (third part). The half circumference of the side on which the tumor is located is defined as 14t, and the half circumference on the opposite side of the side on which the tumor is located is defined as 14op.

**FIGURE 3:**
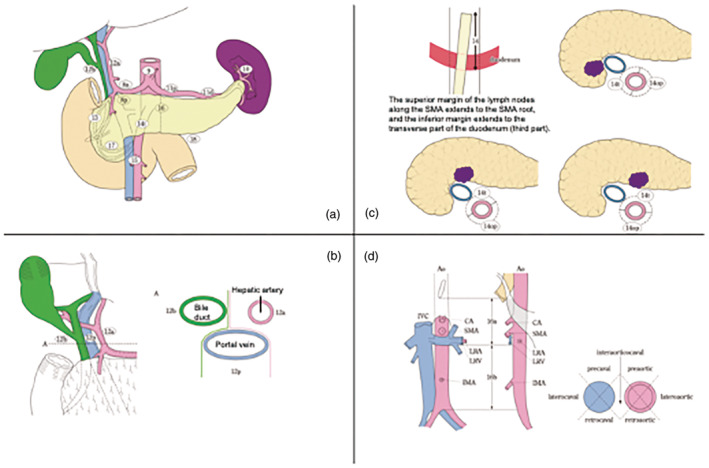
Station numbers and locations of lymph nodes. Reproduced with permission from the Japan Pancreas Society and Kanehara & Co., Ltd.: Japanese Classification of Pancreatic Carcinoma, eighth edition, 2023. Station numbers of lymph nodes in relation to the pancreas. (a) The location and boundaries of the lymph nodes within the hepatoduodenal ligament. The diagram on the right shows the transverse view at line A in the diagram on the left (b), with details provided along the superior mesenteric artery (c) and around the aorta (d). Ao, aorta; CA, celiac artery; IMA, inferior mesenteric artery; IVC, inferior vena cava; LRA, left renal artery; LRV, left renal vein; SMA, superior mesenteric artery.

#### 3.2.2 Regional lymph nodes

Regional lymph nodes are not synonymous with lymph nodes to be dissected. If metastasis is found in a lymph node other than a regional lymph node, it is defined as M1 (Table 2). In the pancreatic head and body, lymph node 14 (tumor side; 14 t) is considered a regional lymph node; however, if metastasis is found in lymph node 14 (tumor opposite side; 14op), it is not considered M1 but instead warrants further investigation in future studies.

**TABLE 2:** Regional lymph nodes.

	Head	Body	Tail
Regional lymph nodes	6, 8a, 8p, 12a, 12b, 12p, 13, 14t, 17	8a, 8p, 9, 10, 11p, 11d, 14t, 18	8a, 9, 10, 11p, 11d, 18

*Note*: Reproduced with permission from the Japan Pancreas Society and Kanehara & Co., Ltd.: Japanese Classification of Pancreatic Carcinoma, eighth edition, 2023.

#### 3.2.3 Recording lymph node metastases

In resected cases, the number of dissected and metastatic nodes should be recorded for each lymph node.

- NX: Regional lymph nodes cannot be assessed
- N0: No regional lymph node metastasis
- N1: Regional lymph node metastasis
  - N1a: Metastasis in one to three regional lymph nodes
  - N1b: Metastasis in four or more regional lymph nodes

### 3.3 Distant metastases (M category)

- M0: No distant metastasis
- M1: Distant metastasis

The M1 site should be recorded using the following codes:

**Table:** 

PUL: pulmonary	MAR: bone marrow	OSS: osseous	PLE: pleura
HEP: hepatic	PER: peritoneum	BRA: brain	ADR: adrenals
LYM: lymph nodes	SKI: skin	PCY: peritoneal cytology	OTH: others

Lymph node metastasis outside the regional nodes should be recorded as M1.

The MX (metastasis cannot be assessed) category is deemed inappropriate because clinical evaluation of distant metastases is possible only through imaging. Use of the MX category may disallow staging.

Specifically, peritoneal, hepatic metastases, and peritoneal cytology positive for carcinoma cells classified as M1 should be recorded as follows:

- Peritoneal metastasis
  - P0: No peritoneal metastasis
  - P1: Peritoneal metastasis
- Hepatic metastasis
  - H0: No hepatic metastasis
  - H1: Hepatic metastasis
- Recording peritoneal cytology
  - CYX: Peritoneal cytology not performed
  - CY0: Peritoneal cytology negative for carcinoma cells
  - CY1: Peritoneal cytology positive for carcinoma cells

The CY1 category is defined as M1 in this classification.

CY1 is recorded when carcinoma cells are detected in ascites fluid and peritoneal lavage.

R1 (residual tumor grading) is recorded when patients with CY1 undergo surgical resection.

CY1 after neoadjuvant therapy is recorded as “ypCY0/1” and defined as M1.

For cytological methods, please refer to Section 5.3, “Procedure for performing peritoneal washing cytology.”

### 3.4 Staging

The JPS staging system aims to aid clinicians in determining appropriate treatment approaches for each stage (Table 3). Generally, stages I and II encompass initially resectable pancreatic cancers. Stage III denotes borderline resectable or locally advanced pancreatic cancer for which neoadjuvant therapy may be recommended. Stage IV is characterized by distant metastasis, warranting systemic chemotherapy as the recommended course of action.

**TABLE 3:** TNM staging.

Stage	T‐category	N‐category	M‐category
Stage 0	T0, Tis	N0	M0
Stage IA	T1 (T1a, T1b, T1c)	N0	M0
Stage IB	T2	N0	M0
Stage IIA	T3	N0	M0
Stage IIB	T0, Tis, T1 (T1a, T1b, T1c), T2, T3	N1 (N1a, N1b)	M0
Stage III	T4	Any N	M0
Stage IV	Any T	Any N	M1

*Note*: Reproduced with permission from the Japan Pancreas Society and Kanehara & Co., Ltd.: Japanese Classification of Pancreatic Carcinoma, eighth edition, 2023.

### 3.5 Resectability classification

#### 3.5.1 Resectable: R

No tumor contact with the superior mesenteric vein (SMV) or portal vein (PV), less than 180 contacts, or invasion without occlusion. The clear fat planes around the SMA, CA, and common hepatic artery (CHA) show no contact or invasion.

#### 3.5.2 Borderline resectable: BR

Subclassified according to SMV/PV invasion alone or arterial invasion.

- BR‐PV (SMV/PV invasion alone): No findings of contact or invasion of the SMA, CA, or CHA. Tumor contact or invasion of the SMV/PV of 180°degrees or more, or occlusion of the SMV/PV, not exceeding the inferior border of the duodenum.
- BR‐A (arterial invasion): Tumor contact or invasion of the SMA and/or CA of less than 180 degrees without stenosis or deformity. Tumor contact or invasion of the CHA without tumor contact or invasion of the PHA and/or CA.

#### 3.5.3 Unresectable: UR

Subclassified according to the status of distant metastasis.

- UR‐LA (locally advanced): tumor contact or invasion of the SMV/PV of 180° or more, or occlusion of the SMV/PV extending beyond the inferior border of the duodenum. Tumor contact or invasion of the SMA and/or CA of 180°or more. Tumor contact or invasion of the CHA, showing tumor contact or invasion of the PHA and/or CA. Tumor contact or invasion of the aorta.
- UR‐M (tumor with distant metastasis): distant metastasis, including nonregional lymph node metastasis.

## 4 SURGICAL TREATMENT

### 4.1 Types of operative procedures

#### 4.1.1 Surgery with or without resection

- Pancreatic resection
- Palliative operation (bypass operations, such as cholangiojejunostomy and gastrojejunostomy)
- Others (including exploratory laparotomy and staging laparoscopy)

#### 4.1.2 Surgical approaches

- Open surgery (O)
- Hand‐assisted laparoscopic surgery (HALS)
- Laparoscopic (L)
- Robot‐assisted (R)
- Others

### 4.2 Description of pancreatic resection

#### 4.2.1 Type of pancreatic resection

Table 4.

**TABLE 4:** Types of pancreatic resections.

Pancreatic head resection (PHR)	Pancreatoduodenectomy (PD)
	Pylorus‐preserving pancreatoduodenectomy (PPPD)
	Subtotal stomach‐preserving pancreatoduodenectomy (SSPPD)
	Duodenum‐preserving pancreatic head resection (DPPHR)
	Pancreatic head resection with segmental duodenectomy (PHRSD)
	Other pancreatic head resection
Distal pancreatectomy (DP)	Distal pancreatectomy (tail)
	Distal pancreatectomy (body‐tail)
	Distal pancreatectomy (subtotal)
	Spleen‐preserving distal pancreatectomy (SPDP)
	Distal pancreatectomy with en‐bloc celiac axis resection (DP‐CAR)
Total pancreatectomy (TP)	Total pancreatectomy (TP)
	Pylorus‐preserving total pancreatectomy (PPTP)
	Spleen‐preserving total pancreatectomy (SPTP)
	Pylorus‐preserving, spleen‐preserving total pancreatectomy (PPSPTP)
	Duodenum‐preserving total pancreatectomy (DPTP)
	Total pancreatectomy with segmental duodenectomy (TPSD)
Middle pancreatectomy (MP)	
Middle segment‐preserving pancreatectomy (MSPP)	
Partial pancreatectomy (PP)	
Enucleation (EN)	

*Note*: Reproduced with permission from the Japan Pancreas Society and Kanehara & Co., Ltd.: Japanese Classification of Pancreatic Carcinoma, eighth edition, 2023.

#### 4.2.2 Combined resection

When combined resection of the duodenum, stomach, colon, spleen, portal system, or artery is performed, the name of each resected organ should be recorded.

#### 4.2.3 Type of reconstruction

##### Order of anastomosis

Reconstructions after pancreatoduodenectomy, pylorus‐preserving pancreatoduodenectomy, or subtotal stomach‐preserving pancreatoduodenectomy are classified and should be recorded according to the order in which the pancreas, bile duct, and stomach are anastomosed to the jejunum, starting from the proximal end of the jejunum (Table 5).

**TABLE 5:** Order of reconstruction.

	Anastomotic order	Description
Type I	Bile duct – pancreas – stomach	PD‐I, PPPD‐I, SSPPD‐I
Type II	Pancreas – bile duct – stomach	PD‐II, PPPD‐II, SSPPD‐II
Type IIIa	Stomach – pancreas – bile duct	PD‐III, PPPD‐III, SSPPD‐III
Type IIIb	Stomach – bile duct – pancreas	PD‐III, PPPD‐III, SSPPD‐III
Type IV	Other anastomoses	PD‐IV, PPPD‐IV, SSPPD‐IV

*Note*: Reproduced with permission from the Japan Pancreas Society and Kanehara & Co., Ltd.: Japanese Classification of Pancreatic Carcinoma, eighth edition, 2023.

##### Reconstruction of the pancreas

- Pancreatojejunostomy
- Pancreatogastrostomy
- Pancreatoduodenostomy
- End‐to‐end anastomosis of the pancreas

These anastomoses are further subclassified into duct‐to‐mucosa, invagination, and dunking technique used, or others.

### 4.3 Lymph node dissection

The stations and numbers of the dissected lymph nodes should be recorded.

### 4.4 Assessment of residual tumor

After pancreatic resection, which includes the primary cancer, the gross or histological evidence of residual tumor (R) is classified as follows:

- RX: Presence of residual tumor cannot be assessed
- R0: No residual tumor
- R1: Microscopic residual tumor
- R2: Macroscopic residual tumor

For R0, the shortest distance (mm) from the cut end margin to the invasion site should be recorded. For localized residual tumors, the presence or absence of invasion at the cut end and dissected margins should be recorded as follows:

#### 4.4.1 Pancreatic cut end margin (PCM)

- PCM0: No cancer infiltration
- PCM1: Cancer infiltration present*
- PCMX: Cancer infiltration cannot be assessed

#### 4.4.2 Bile duct cut end margin (BCM)

- BCM0: No cancer infiltration
- BCM1: Cancer infiltration present*
- BCMX: Cancer infiltration cannot be assessed

*PCM1/BCM1 with carcinoma in situ only is recorded as PCM/BCM1e (epithelium), whereas PCM1/BCM1 with invasive carcinoma or both cancer in situ and invasive carcinoma is recorded as PCM1/BCM1i (invasive).

#### 4.4.3 Dissected peripancreatic tissue margin (DPM)

- DPM0: No cancer infiltration
- DPM1: Cancer infiltration present
- DPMX: Cancer infiltration cannot be assessed

### 4.5 Conversion surgery

Conversion surgery, a term that originated in Japan, is a surgical resection procedure for patients with unresectable pancreatic cancer (locally advanced: UR‐LA, distant metastasis: UR‐M) diagnosed initially and whose disease is controlled after a certain period of nonoperative treatment (multidisciplinary treatment). With the development of multidisciplinary treatments for pancreatic cancer, the number of cases converting from nonoperative therapy to surgical therapy has been increasing.

## 5 HANDLING OF RESECTED SPECIMENS

### 5.1 Handling of resected pancreatic specimens

**FIGURE 4:**
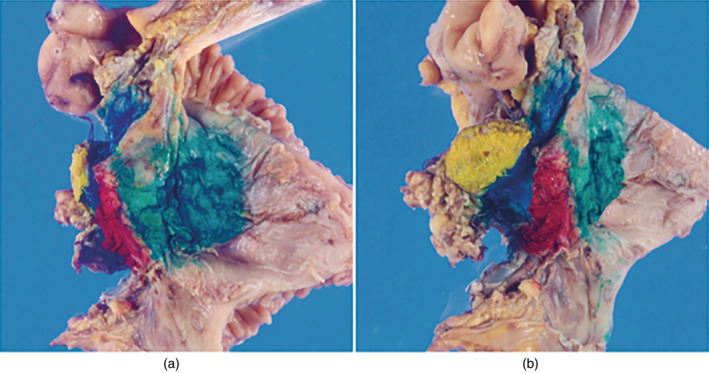
Inking example. Reprinted with permission from the Japan Pancreas Society and Kanehara & Co., Ltd.: Japanese Classification of Pancreatic Carcinoma, eighth edition, 2023. Pancreatoduodenectomy specimen after fixation. (a) View from the posterior surface. (b) View from the transection margin (blue, portal vein margin; red, superior mesenteric artery margin; green, posterior surface; yellow, transection margin).

The specimen should be examined grossly from both the dorsal and abdominal sides. If a tumor involves the pancreatic capsule or a desquamated surface, its location, size, and appearance should be recorded. The margins should be inked, and the most invasive site should be sampled (Figure 4).

For use in comprehensive genomic profiling and companion diagnostics, in addition to routine pathology, specimens are immediately immersed in a sufficient volume (approximately 10 times the tissue volume) of 10% neutral‐buffered formalin solution and fixed for 6–48 h at room temperature.

### 5.2 Sectioning of the specimen

- Pancreatoduodenectomy specimens: Kerckring's fold line, which passes through the aperture of the papilla of Vater, is used as the reference line. The specimen should be serially sectioned parallel to the reference line at approximately 5‐mm intervals.
- Distal pancreatectomy specimens: The specimen should be serially sectioned perpendicular to the long axis of the pancreas at approximately 5‐mm intervals.
- Total pancreatectomy specimens: The specimen should be sectioned by combining the procedures described in pancreatoduodenectomy and distal pancreatectomy.

### 5.3 Procedure for performing peritoneal washing cytology

Immediately after laparotomy, if ascites fluid is present, it is collected and examined; otherwise, the peritoneal cavity is gently washed with 100 mL of physiological saline, and the fluid is collected from the rectovesical pouch or Douglas pouch and examined. Subsequently, the ascites or washing fluid is centrifuged, and the sediment is smeared onto a glass slide. The addition of anticoagulants is not recommended except in the case of severe bloody ascites. Papanicolaou staining is the basic stain, and at least two other stains, such as Giemsa staining, are recommended. If necessary, immunostaining or other special stains may be added.

## 6 HISTOLOGICAL FINDINGS OF PANCREATIC TUMORS

### 6.1 Histological classification of pancreatic tumors

Table 6.

**TABLE 6:** Histological classification of pancreatic tumors.

[1] Epithelial neoplasms
A. Exocrine neoplasms
1. Serous neoplasms (SNs)
(a) Serous cystadenoma (SCA)
(b) Serous cystadenocarcinoma (SCC)
2. Mucinous cystic neoplasms (MCNs)
(a) Mucinous cystadenoma (MCA)
(b) Mucinous cystadenocarcinoma (MCC), noninvasive
(c) Mucinous cystadenocarcinoma (MCC), invasive
3. Intraductal neoplasms
(a) Intraductal papillary mucinous neoplasms (IPMNs)
(1) Intraductal papillary mucinous adenoma (IPMA)
(2) Intraductal papillary mucinous carcinoma (IPMC), noninvasive
(3) Intraductal papillary mucinous carcinoma (IPMC), invasive
(b) Intraductal oncocytic papillary neoplasms (IOPNs)
(1) Intraductal oncocytic papillary carcinoma (IOPC), noninvasive
(2) Intraductal oncocytic papillary carcinoma (IOPC), invasive
(c) Intraductal tubulopapillary neoplasms (ITPNs)
(1) Intraductal tubulopapillary carcinoma (ITPC), noninvasive
(2) Intraductal tubulopapillary carcinoma (ITPC), invasive
(d) Pancreatic intraepithelial neoplasia (PanIN)
(1) Low‐grade PanIN
(2) High‐grade PanIN
4. Invasive ductal carcinomas (IDCs)
(a) Adenocarcinoma^a^
(i) Well differentiated type (wel)
(ii) Moderately differentiated type (mod)
(iii) Poorly differentiated type (por)
(b) Adenosquamous carcinoma (asc)
(c) Mucinous carcinoma (muc)
(d) Anaplastic carcinoma (anc)^b^
(i) Anaplastic carcinoma, pleomorphic type
(ii) Anaplastic carcinoma, spindle cell type
(iii) Anaplastic carcinoma with osteoclast‐like giant cells
5. Acinar cell neoplasms (ACNs)
(a) Acinar cystic transformation (ACT)
(b) Acinar cell carcinoma (ACC)
B. Neuroendocrine neoplasms (NENs)
1. Neuroendocrine tumors (NETs, G1, G2, G3)
2. Neuroendocrine carcinoma (NEC)
C. Mixed neoplasms/mixed neuroendocrine non‐neuroendocrine neoplasms (MiNENs)
D. Epithelial neoplasms of uncertain differentiation
1. Solid pseudopapillary neoplasm (SPN)
2. Pancreatobalstoma
E. Unclassifiable
F. Miscellaneous
[2] Nonepithelial neoplasms
Hemangioma, lymphangioma, leiomyosarcoma, malignant lymphoma, paraganglioma, others

*Note*: Reproduced with permission from the Japan Pancreas Society and Kanehara & Co., Ltd.: Japanese Classification of Pancreatic Carcinoma, eighth edition, 2023.

^a^ Adenocarcinoma is subcategorized into well differentiated type, moderately differentiated type, and poorly differentiated type according to the degree of duct formation (histological differentiation) of the predominant component.

^b^ Anaplastic carcinoma has unclear differentiation and generally corresponds to undifferentiated carcinoma defined by the WHO Classification (fifth edition).

### 6.2 Cancer‐stroma relationship

Tumors should be classified into the following types according to the proportion of stroma they contain:

- Medullary type (med): Tumors containing scanty stroma.
- Intermediate type (int): Tumors containing a proportion of stroma intermediate between scirrhous and medullary types.
- Scirrhous type (sci): Tumors containing abundant stroma.

### 6.3 Growth patterns of neoplasms infiltrating the surrounding tissue

The most dominant growth patterns at the neoplasm margin are as follows:

- INFa: The tumor shows expanding growth, with a border distinct from the surrounding tissues.
- INFb: An intermediate growth between INFa and INFc.
- INFc: The tumor shows infiltrating growth with a border indistinct from the surrounding tissue.

### 6.4 Lymphatic invasion

- Ly0: No invasion
- Ly1a: Slight invasion
- Ly1b: Moderate invasion
- Ly1c: Marked invasion

### 6.5 Venous invasion

- V0: No invasion
- V1a: Slight invasion
- V1b: Moderate invasion
- V1c: Marked invasion

### 6.6 Perineural/neural invasion

- Pn0: No invasion
- Pn1a: Slight invasion
- Pn1b: Moderate invasion
- Pn1c: Marked invasion

### 6.7 Main pancreatic ductal spread beyond the area of invasive cancer

- mpd0: Absent
- mpd1: Present
- mpdx: Undetermined

## 7 BIOPSY/CYTOLOGY OF PANCREATIC NEOPLASMS

### 7.1 Reporting system for endoscopic ultrasound‐guided fine needle aspiration biopsy of pancreatic lesions

**FIGURE 5:**
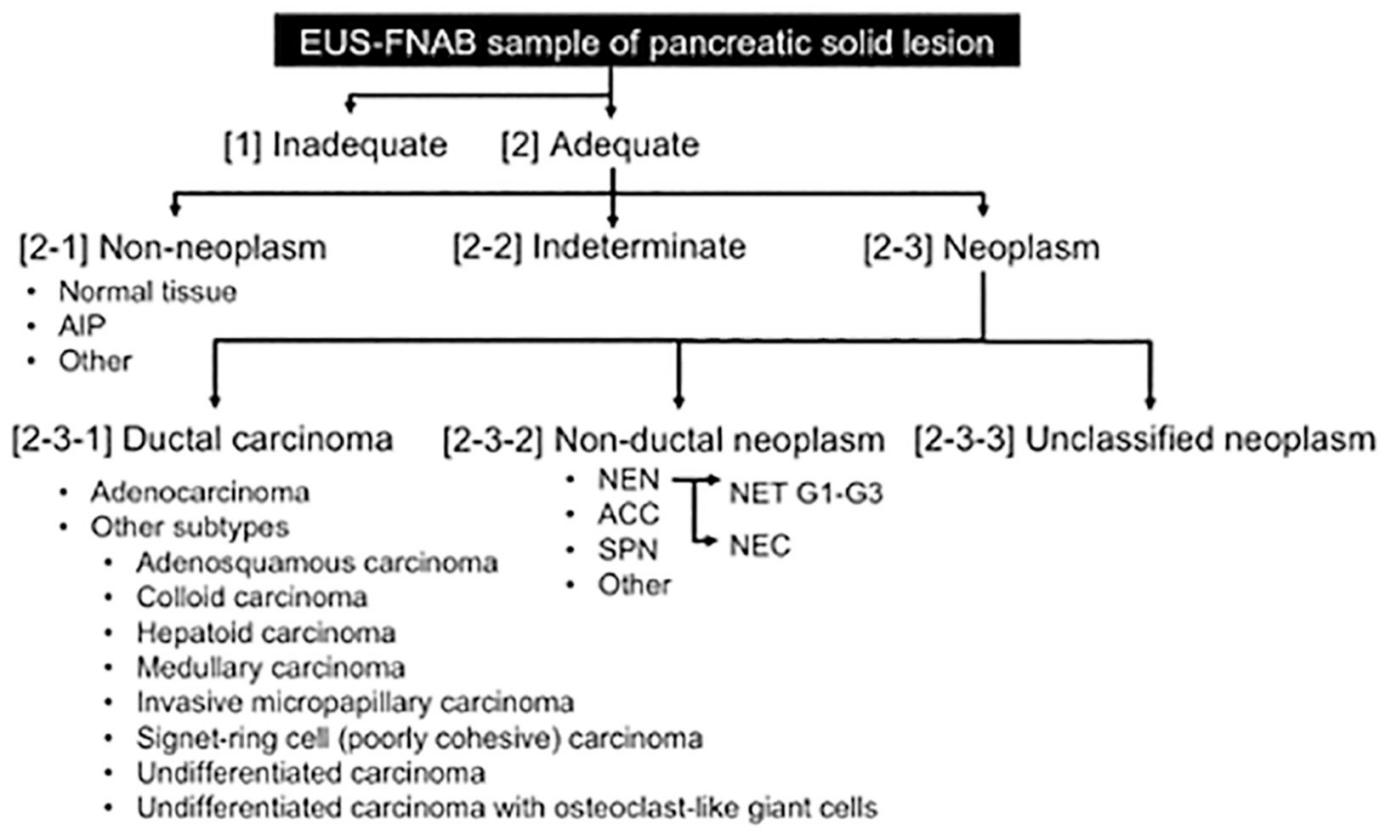
Diagnostic categories of EUS‐FNAB samples of pancreatic solid lesions. Adapted from reference.^8^ [1] Inadequate: This category should be applied to specimens that are not suitable for diagnosis due to insufficient quantity or quality. The reasons for unsuitability, such as degeneration or artifacts, should be noted. [2] Adequate: This category should be applied to specimens suitable for diagnosis. The diagnostic procedures should be evaluated according to the following subcategories: Non‐neoplasm, indeterminate, and neoplasm. Whether the sample size is large or small should be assessed. [2‐1] Non‐neoplasm: This subcategory should be applied to specimens with non‐neoplastic lesions. Specimens suggestive of autoimmune pancreatitis can be further evaluated by elastica staining and immunohistochemistry for IgG4. [2‐2] Indeterminate: This subcategory should be applied to specimens that have sufficient quantity and quality but present challenges in evaluation. This subcategory may include specimens that are suggestive of malignancy but pose challenges in definitive determination. The term “suggestive of malignancy” should be employed in consultation with local clinicians to guide management decisions. Samples in this subcategory can be further evaluated using stepwise sections and/or immunohistochemistry. [2‐3] Neoplasm: This subcategory should be applied to specimens evaluated as neoplastic lesions, which should be further categorized into the following diagnostic categories, including ductal carcinoma, nonductal neoplasm, and unclassified neoplasm. [2‐3‐1] Ductal carcinoma: This diagnostic category should be applied to specimens sufficiently evaluated for ductal carcinoma according to the histological features listed in Table 7. Acinar ductal metaplasia can be differentiated using immunohistochemical staining for p53 and maspin. [2‐3‐2] Non‐ductal neoplasm: This diagnostic category should be applied to specimens sufficiently evaluated as nonductal neoplasms, such as neuroendocrine neoplasms, acinar cell carcinomas, solid pseudopapillary neoplasms, metastatic neoplasms, and nonepithelial neoplasms. Immunohistochemical analysis may be useful for this evaluation. [2‐3‐3] Unclassified neoplasm: This diagnostic category should be applied to specimens evaluated as neoplasms with an indeterminate ductal nature according to further immunohistochemical examinations. ACC, acinar cell carcinoma; AIP, autoimmune pancreatitis, EUS‐FNAB, endoscopic ultrasound‐guided fine needle aspiration biopsy; NEC, neuroendocrine carcinoma; NEN, neuroendocrine neoplasm; NET, neuroendocrine tumor; SPN, solid pseudopapillary neoplasm.

Endoscopic ultrasound‐guided fine needle aspiration biopsy (EUS‐FNAB) of pancreatic lesions is a routine procedure in Japan. It is clinically imperative to histopathologically diagnose solid pancreatic lesions. However, EUS‐FNAB is generally not recommended for cystic lesions because of the risk of fluid content leakage. Under this guidance, the following reporting system was proposed to ensure accurate and reliable communication with clinicians (Figure 5, Table 7).^8^

**TABLE 7:** Histological features relevant for the detection of EUS‐FNAB samples of PDAC. Reproduced from reference.^8^

1. Overall low power appearance	Necrotic debris in the background
	Abundant detached atypical cells in the necrotic debris or clot
	Numerous sheets of atypical cells
2. Structural atypia/abnormalities	Irregular contour (ex. cribriform pattern)
	Lack of uniformity of glandular formation
	Luminal necrosis
3. Cellular atypia	Enlarged nuclei
	Irregular nuclear contour
	Prominent nucleoli
	Hyperchromatism
	Anisonucleosis 4:1 within a single duct
	Morphological heterogeneity of intracellular mucus
	Easily identifiable mitotic figures
	Clear or foamy cell change in the cytoplasm
4. Parenchymal invasion	Haphazard (or irregular) arrangement of glands
	Stromal desmoplasia

### 7.2 Pancreatic cytology reporting system

The cytology reporting system in this classification follows the previous version of Classification of Pancreatic Carcinoma by the JPS.^3^ This system is consistent with the Papanicolaou Society of Cytopathology 2014 Guideline^9^ and the Atlas and Guideline for Cytopathological Diagnosis 5 of the Japanese Society of Clinical Cytology for the pancreatic region.^10^

#### 7.2.1 Reporting format and evaluation

The first step is to evaluate the appropriateness of the specimens (Table 8). Thus, the number of cells must be adequate for pancreatic juice specimens. When brushing the specimens, the sample should not be dry. For specimens obtained via EUS‐FNAB, the presence of a tumor should be noted. The decision should focus on benign or malignant lesions, and the detailed cytological findings and probable lesions should be described. In cases where diagnosis is difficult, indicating whether the lesion is benign or malignant is recommended.

**TABLE 8:** Reporting format and evaluation.

1. Classification	Inadequate		
	Adequate	Negative/benign	
		Atypical/indeterminate	Favor benign
			Favor malignant
			Others
		Suspicious for malignancy/at least low‐grade malignancy	
		Positive/malignant	
2. Description of findings (e.g., grade of atypia), or presumable diagnosis			

*Note*: Reproduced with permission from the Japan Pancreas Society and Kanehara & Co., Ltd.: Japanese Classification of Pancreatic Carcinoma, eighth edition, 2023.

#### 7.2.2 Classification of intraoperative peritoneal cytology

- CYX: Peritoneal cytology is not performed
- CY0: Peritoneal cytology is negative for carcinoma cells
- CY1: Peritoneal cytology is positive for carcinoma cells

## 8 HISTOLOGICAL ASSESSMENT OF PREOPERATIVE THERAPEUTIC EFFECTS

Chemotherapy, molecular‐targeted therapy, and radiation therapy can induce changes in pancreatic cancer tissues (Table 9). The histological response of pancreatic cancer is determined based on the degree of these changes. The current method for determining treatment efficacy is based on the percentage of remaining viable cancer cells. Cells that exhibit nuclear enrichment (pyknosis), karyorrhexis, karyolysis, or nuclear loss are considered nonviable. Stromal findings, such as xanthogranuloma‐like appearances and mucus pools, may help assess the treatment response. However, estimating the pre‐existing tumor area is challenging. Only changes in invasive lesions in posttreatment pancreatectomy specimens are evaluated. In cases where determining the grade is challenging, the less effective one should be selected. Currently, the College of American Pathologists (CAP) classification is widely used to determine the residual tumor volume without considering the volume or extent of the tumor prior to treatment. However, its use remains controversial. Currently, a combination of the JPS and CAP classifications is preferred.

**TABLE 9:** Histological assessment of preoperative therapeutic effects.

Grade 1: Poor or no response: The therapeutic response is poor (estimated residual cancer rate ≧ 50%)
Grade 1a: Estimated residual cancer rate ≧90%
Grade 1b: 50% ≦ estimated residual cancer rate <90%
Grade 2: Moderate response: A moderate amount of viable cancer cells is found (10% ≦ estimated residual rate <50%)
Grade 3: Marked response: Only a small amount of viable cancer cells is found (estimated residual rate <10%)
Grade 4: Complete response: No viable cancer cells are found

*Note*: Reproduced with permission from the Japan Pancreas Society and Kanehara & Co., Ltd.: Japanese Classification of Pancreatic Carcinoma, eighth edition, 2023.

## CONFLICT OF INTEREST STATEMENT

The authors declare there are no conflicts of interest.
